# The actin-myosin regulatory MRCK kinases: regulation, biological functions and associations with human cancer

**DOI:** 10.1007/s00109-014-1133-6

**Published:** 2014-02-20

**Authors:** Mathieu Unbekandt, Michael F. Olson

**Affiliations:** Cancer Research UK Beatson Institute, Switchback Road, Garscube Estate, Glasgow, UK G61 1BD

**Keywords:** Actin, Myosin, Cytoskeleton, MRCK, Kinase, Cancer

## Abstract

The contractile actin-myosin cytoskeleton provides much of the force required for numerous cellular activities such as motility, adhesion, cytokinesis and changes in morphology. Key elements that respond to various signal pathways are the myosin II regulatory light chains (MLC), which participate in actin-myosin contraction by modulating the ATPase activity and consequent contractile force generation mediated by myosin heavy chain heads. Considerable effort has focussed on the role of MLC kinases, and yet the contributions of the myotonic dystrophy-related Cdc42-binding kinases (MRCK) proteins in MLC phosphorylation and cytoskeleton regulation have not been well characterized. In contrast to the closely related ROCK1 and ROCK2 kinases that are regulated by the RhoA and RhoC GTPases, there is relatively little information about the CDC42-regulated MRCKα, MRCKβ and MRCKγ members of the AGC (PKA, PKG and PKC) kinase family. As well as differences in upstream activation pathways, MRCK and ROCK kinases apparently differ in the way that they spatially regulate MLC phosphorylation, which ultimately affects their influence on the organization and dynamics of the actin-myosin cytoskeleton. In this review, we will summarize the MRCK protein structures, expression patterns, small molecule inhibitors, biological functions and associations with human diseases such as cancer.

## Introduction

The actin-myosin cytoskeleton acts as the internal scaffold that determines cell shape. Through the co-ordinated elongation and contraction of actin-myosin filaments and meshes, force may be generated to power activities such as changes in cell morphology, division and motility. There are numerous ways that actin-myosin dynamics are regulated, but one of the key events in non-muscle cells is the phosphorylation of the myosin II regulatory light chain (MLC) [1]. MLC phosphorylation on Ser19, either alone or in combination with Thr18 phosphorylation, regulates contractility by influencing the ATPase activity of the myosin heavy chain (MHC) head groups, which require energy from ATP-hydrolysis to “walk” along actin filaments to produce contractile force. The phosphorylation status of MLC at any given moment is the product of kinase and phosphatase activities. Although numerous kinases influence MLC phosphorylation status, in some cases this may be mediated through the inactivation of MLC phosphatase activity rather than via direct MLC phosphorylation [2]. Western blotting is often used to measure changes in bulk MLC phosphorylation; however, small changes in spatially restricted MLC phosphorylation are very important for the regulation of actin-myosin cytoskeleton dynamics that affect processes such as cell adhesion, morphology and motility.

There are two major signalling pathways that lead to MLC phosphorylation, either Ca^2+^ mobilization to activate calcium-calmodulin dependent myosin light chain kinases [2] or Rho GTPase activation leading to stimulation of several downstream effector kinases [1]. For example, activation of RhoA and RhoC lead to increased ROCK1 and ROCK2 kinase activity [3], as well as stimulation of the citron rho-interacting kinase (CRIK) that has specialized functions during cytokinesis [4]. The contributions of ROCK1 and ROCK2 to MLC phosphorylation and consequent biological responses have been extensively studied in numerous contexts, aided in no small part by the development of potent and reasonably selective small molecule inhibitors such as Y27632 [5, 6]. In addition to ROCK and CRIK, the Myotonic dystrophy-related Cdc42-binding kinases (MRCK) α [7], MRCKβ [7] and MRCKγ [8] contribute to MLC phosphorylation downstream of CDC42. However, much less is known about the relative importance of MRCK in actin-myosin regulation, or their roles in cell biology. By summarizing the knowledge about MRCK, we hope to encourage consideration of how these kinases may be important contributors to the regulation of actin-myosin cytoskeletal dynamics and how they may be involved in human diseases such as cancer.

## MRCK are multi-domain AGC family kinases

**Fig. 1:**
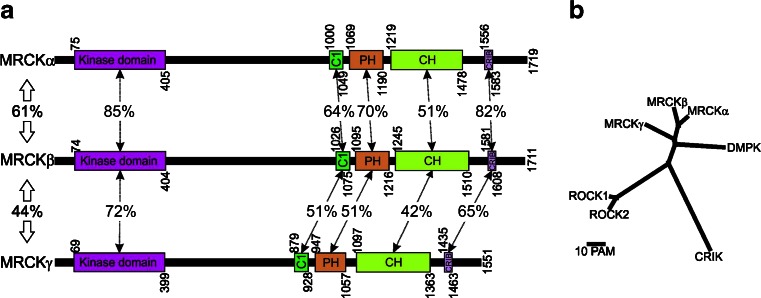
Homology between MRCK proteins and related kinases. **a** Protein domains and their indicated positions were taken from the National Center for Biotechnology Information (NCBI; http://www.ncbi.nlm.nih.gov/protein) for human MRCKα (NP_003598.2), MRCKβ (NP_006026.3) and MRCKγ (NP_059995.2). Percentage amino acid identities were determined with the Basic Local Alignment Search Tool (BLAST; http://blast.ncbi.nlm.nih.gov/Blast.cgi). *C1* protein kinase C conserved region 1, *PH* Pleckstrin homology-like, *CH* citron homology, *CRIB* CDC42/Rac interactive binding. **b** Multiple sequence alignment with hierarchical clustering (http://multalin.toulouse.inra.fr/multalin) was used to create a phylogenetic tree showing the evolutionary relatedness of the kinase domains from MRCK and close homologues. Distance between proteins is depicted by the *scale bar*, where PAM units are a function of random mutations during evolution that generate diversity

The three MRCK proteins are serine/threonine kinases that are part of the AGC (PKA, PKG and PKC) kinase family [9]. The human MRCKα kinase domain was first discovered in a yeast two-hybrid screen for proteins binding to the intracytoplasmic portion of the granulocyte-macrophage colony-stimulating factor alpha subunit, and was initially given the name PK428 [10]. The MRCKα *Drosophila* homologue Genghis Khan (Gek) was subsequently isolated in yeast two-hybrid screens for proteins binding specifically to active GTP-bound CDC42 but not to inactive GDP-bound CDC42 [11]. Full-length rat MRCKα and MRCKβ were independently identified by an expression cloning screen for proteins that associated with CDC42 bound to ^32^P-labelled GTP followed by probing of a brain cDNA library with the isolated open reading frame fragment [7]. Human MRCKα [12] and MRCKβ [13] were subsequently discovered by a combination of RT-PCR using degenerate oligonucleotide primers and DNA database searches. MRCKγ (172 kDa) was first identified in searches for novel Cdc42/Rac interactive binding (CRIB) domain (Fig. 1a) containing genes [14], and the human open reading frame was subsequently cloned and characterized [8]. To date, no knockout mice for any of the MRCK genes have been reported. Although initially identified on the basis of their binding to GTP-loaded CDC42 [7, 11], the ability of Rac1 to associate with MRCKα suggests that these kinases may also act as effectors in Rac signalling pathways [15]. Further analysis to rigorously measure the affinities of MRCK CRIB domains for GTP-bound CDC42 and Rac1, as well as unbiased proteomics-based identification of associated proteins would help determine how significantly MRCK proteins act as CDC42 and/or Rac effectors.

Multiple sequence alignment with hierarchical clustering [16] to analyse the homology between kinase domains and phylogenetic relationships (Fig. 1b) revealed that MRCKα and MRCKβ are very closely related with 85 % amino acid identity (Fig. 1a), while MRCKγ is the nearest additional homologue with 72 % identity relative to MRCKβ. The Dystrophia Myotonica Protein Kinase (DMPK) is on a separate branch from the 3 MRCK proteins, while the ROCK1 and ROCK2 kinases are more distantly related (Fig. 1b). The crystal structure of the MRCKβ kinase domain indicated that it forms dimers and revealed the close conservation of three-dimensional spatial organization of this region when compared with the structures of the ROCK1, ROCK2 and DMPK kinase domains [17]. Although it was initially reported that there were important regulatory roles for autophosphorylation of MRCKα on Ser234, Thr240 and Thr403 in the kinase domain [18], the structures of MRCKβ [17], DMPK [19], ROCK1 [20] and ROCK2 [21] kinase domains were all found in active conformations in the absence of phosphorylation, indicating that this post-translational modification is apparently not essential for attainment of the active conformation. The high homology of the MRCK kinase domains compared with the DMPK kinase domain resulted in some monoclonal antibodies raised against DMPK to also bind a conserved epitope on MRCKα and MRCKβ [22]. CRIK has a degree of homology that indicates it is a relative and part of a greater family, but has diverged sufficiently to be separate from MRCK, DMPK and ROCK kinases (Fig. 1b). Interestingly, although the kinase domains are conserved, as are their roles in Rho GTPase signalling pathways, the MRCK kinases act downstream of CDC42/Rac1 via their CRIB domains while ROCK/CRIK proteins have Rho-binding domains to participate in Rho-regulated pathways.

MRCKα and MRCKβ are highly homologous, with 61 % amino acid identity across their entire primary amino acid sequence, while the less closely related MRCKγ has 44 % identity with MRCKβ (Fig. 1a). In addition to their well-conserved kinase domains, all three proteins have protein kinase C conserved region 1 (C1) domains (Fig. 1a), which in the case of MRCKα and MRCKβ have been shown to bind phorbol esters with nanomolar affinities [18, 23]. Phorbol ester binding to the C1 domains may promote kinase activation [18], and/or may contribute to membrane translocation [23]. The relatively lower affinities of MRCK C1 domains for phorbol esters compared to PKC C1 domains and inefficient plasma membrane recruitment of an isolated C1 domain led to the suggestion that additional regions might make important contributions to membrane translocation [23]. The Pleckstrin Homology (PH)-like domains found in all three MRCK proteins (Fig. 1a) are marked by a structural fold similar to that found in archetypal PH domains but without significant primary amino acid identity. The PH-like domains are generally believed to aid in targeting proteins to appropriate subcellular localizations through binding to lipid and/or protein partners. Therefore, it is possible that this domain provides an additional membrane-targeting input or contributes to substrate protein docking.

All three MRCK proteins have a Citron Homology (CH) domain, which derives its name from being found also in CRIK where it is similarly adjacent to a PH-like domain [4]. The conservation of this spatial arrangement of PH-CH domains suggests that they act in tandem and have similar roles in MRCK and CRIK proteins, possibly in mediating protein-protein interactions that specify protein localization or substrate docking. Finally, binding of CDC42-GTP to CRIB domains (Fig. 1b) was the basis for the initial identification of MRCK proteins, although Rac1 binding may also be an important factor [15]. The ability of MRCKα to phosphorylate recombinant MLC in vitro was not increased by the addition of CDC42-GTP [7], suggesting that the primary purpose of the MRCK-CDC42 interaction might be membrane recruitment rather than regulation of kinase specific activity. The array and arrangement of C1-PH-CH-CRIB domains in MRCK carboxyl-termini suggests that this region may act in concert to integrate numerous membrane localization signals, and possibly to specify regions in the membrane with particular lipid/protein composition, to mediate membrane translocation in response to activation of signalling pathways. Consistent with this possibility, treatment of cells from the kidney collecting duct with vasopressin was shown to result in enhanced accumulation of MRCKβ at the apical plasma membranes [24]. The differences between MRCK and ROCK in their non-catalytic regions may be important for directing each protein to different subcellular localizations to provide spatially distinct mechanisms for actin-myosin regulation.

## MRCK expression

**Fig. 2:**
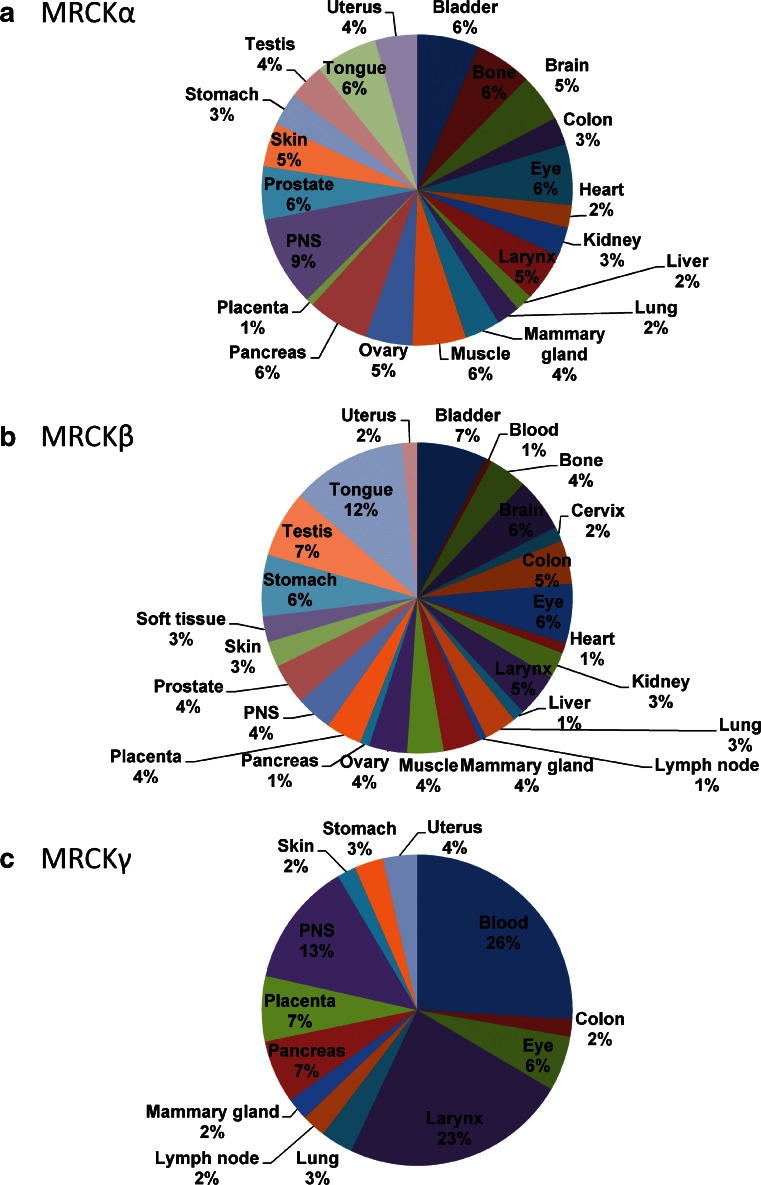
Tissue distribution of MRCK determined from expressed sequence tags (EST). **a** MRCKα, **b** MRCKβ and **c** MRCKγ relative expression levels were derived from the Tissue-specific Gene Expression and Regulation (TiGer) database (http://bioinfo.wilmer.jhu.edu/tiger). The expression levels were normalized with tissue-library size. Each value for a gene in a tissue is a ratio of observed ESTs to the expected one in this tissue. The expected number of ESTs is the product of total ESTs of the gene and the fraction of total ESTs in the tissue among all ESTs in 30 tissues. To depict tissue expression profiles, the normalized expression levels were graphed as percentages from only those tissues having values >0

The three MRCK genes have been reported to give rise to transcripts of varying sizes [8, 13, 18], suggesting that there may be extensive alternative splicing events. However, there has not been a systematic characterization of MRCK alternative splicing to determine how it might affect the proteins produced. MRCKα and MRCKβ were initially reported to be ubiquitously expressed and display their highest expression levels in the brain [7]. Analysis of MRCKα (Fig. 2a) and MRCKβ (Fig. 2b) expressed sequence tag (EST) distribution using the Tissue-specific Gene Expression and Regulation (TiGer) database [25] revealed that their distribution patterns were similar and that no specific organ/tissues had expression levels that were notably higher than another. MRCKγ expression was initially reported to be restricted to the heart and skeletal muscle [8], similar to DMPK expression [22]. However, EST analysis indicates that MRCKγ expression (Fig. 2c) is indeed relatively restricted to fewer tissues, with blood, larynx and peripheral nervous system (PNS) having the highest expression levels. Post-transcriptionally, an iron responsive element has been identified on the 3′UTR of MRCKα that influences mRNA stability in response to iron levels [26]. As is the case for Transferin Receptor 1 mRNA transcripts, MRCKα mRNA is stabilized by low iron levels and destabilized by high iron levels, suggesting that there may be a potential role for MRCKα in iron uptake, or in contributing to the regulation of iron-dependent processes.

## MRCK inhibitors

**Fig. 3:**
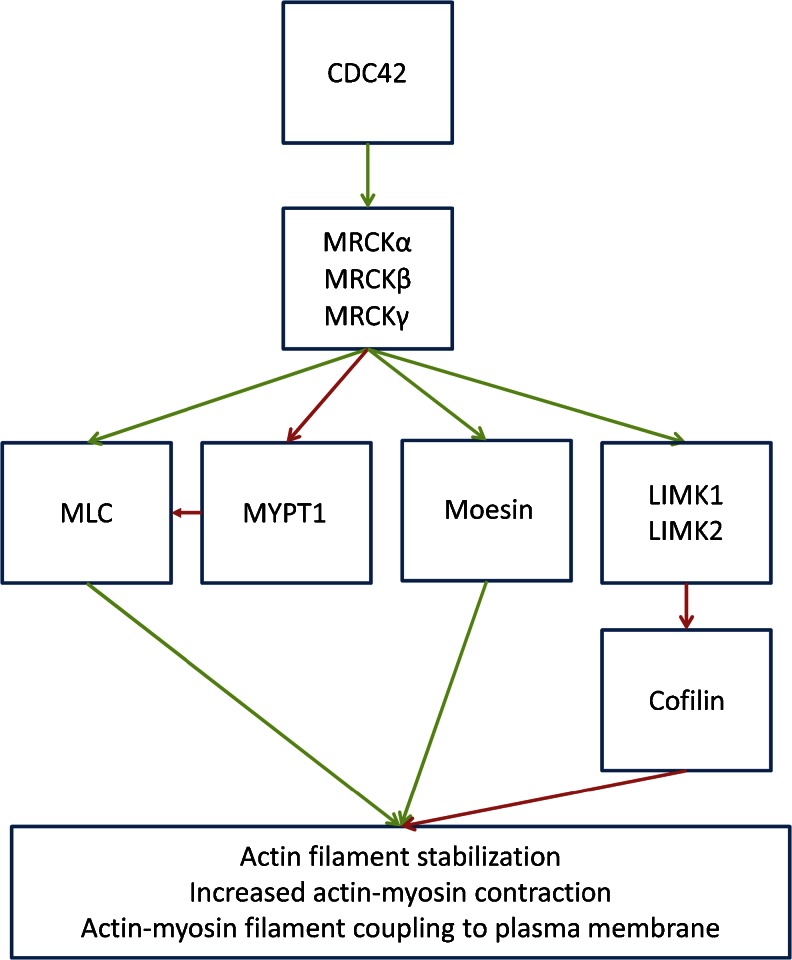
MRCK signalling pathway. The MRCK kinases phosphorylate substrates that may either be activating (*green arrow*) or inactivating (*red arrow*). Phosphorylation of MYPT1 reduces MLC dephosphorylation (*red arrow*), while LIM kinase (*LIMK*) phosphorylation of Cofilin blocks its filamentous actin severing activity (*red arrow*). Two consecutive *red arrows* results in inhibition of a negative activity. The net effect of these events is increased actin-myosin contraction

A major contributing factor to the depth of knowledge about ROCK biological functions is the ready availability of small molecule inhibitors with reasonable potency and selectivity profiles, such as Y27632 [5]. In contrast, there are currently no known selective inhibitors for MRCK. This absence of chemical biology tools is responsible for the lack of information on the role of MRCK in cellular activities that are known to be influenced by ROCK, such as motility, proliferation and survival. The benzophenanthridine chelerythrine (Fig. 3a) was initially identified as a protein kinase C inhibitor with in vitro IC_50_ of 660 nM [27], and was subsequently reported to inhibit MRCKα kinase activity with an in vitro IC_50_ of 1.77 μM [28]. The inhibition of MRCKα by chelerythrine was not affected by raising the ATP concentration as much as 20-fold higher, indicating that the mode of inhibition was unlikely to be via ATP-competition. A lack of selectivity makes chelerythrine difficult to use for cell-based or in vivo experiments to evaluate MRCK function, additional reported off-target effects include reactive oxygen species generation [29], DNA intercalation [30] and inhibition of acetylcholinesterases [31]. By coupling the AKT inhibitor GSK690693 to solid substrates to facilitate purification of interacting proteins, MRCKβ was identified as a major interacting protein [32]. However, it remains to be determined whether GSK690693 inhibits MRCK activity. The natural compound cycloartane 3,24,25-triol (Fig. 3b) was identified as a potential MRCKα inhibitor [33] on the basis of its ability to compete an immobilized ligand for binding to the ATP-binding site [34]. Further characterization is required to determine whether it effectively inhibits MRCK activity in vitro or in cells. By screening a kinase inhibitor chemical library for hits that reduced in vitro catalytic activity, the non-selective kinase inhibitor staurosporine (Fig. 3c), ROCK inhibitors, fasudil, H89 and Y27632 (Fig. 3d), and IκB Kinase 2 inhibitor TCPA-1 [35] were shown to inhibit MRCKβ kinase domain activity in vitro when assays were carried out at the ATP K_i_ of 0.7 μM [17]. Crystals of the MRCKβ kinase domain in complex with fasudil and TCPA-1 were obtained, which revealed how they interact with amino acids lining the ATP-binding pocket [17]. These studies revealed that there are structural features of the MRCK ATP-binding pocket that differ sufficiently from ROCK that it should be possible to develop MRCK-selective inhibitors. However, at this time there do not appear to be potent MRCK inhibitors that could be used as tools to study MRCK activity and functions.

### MRCK kinase substrates

**Fig. 4:**
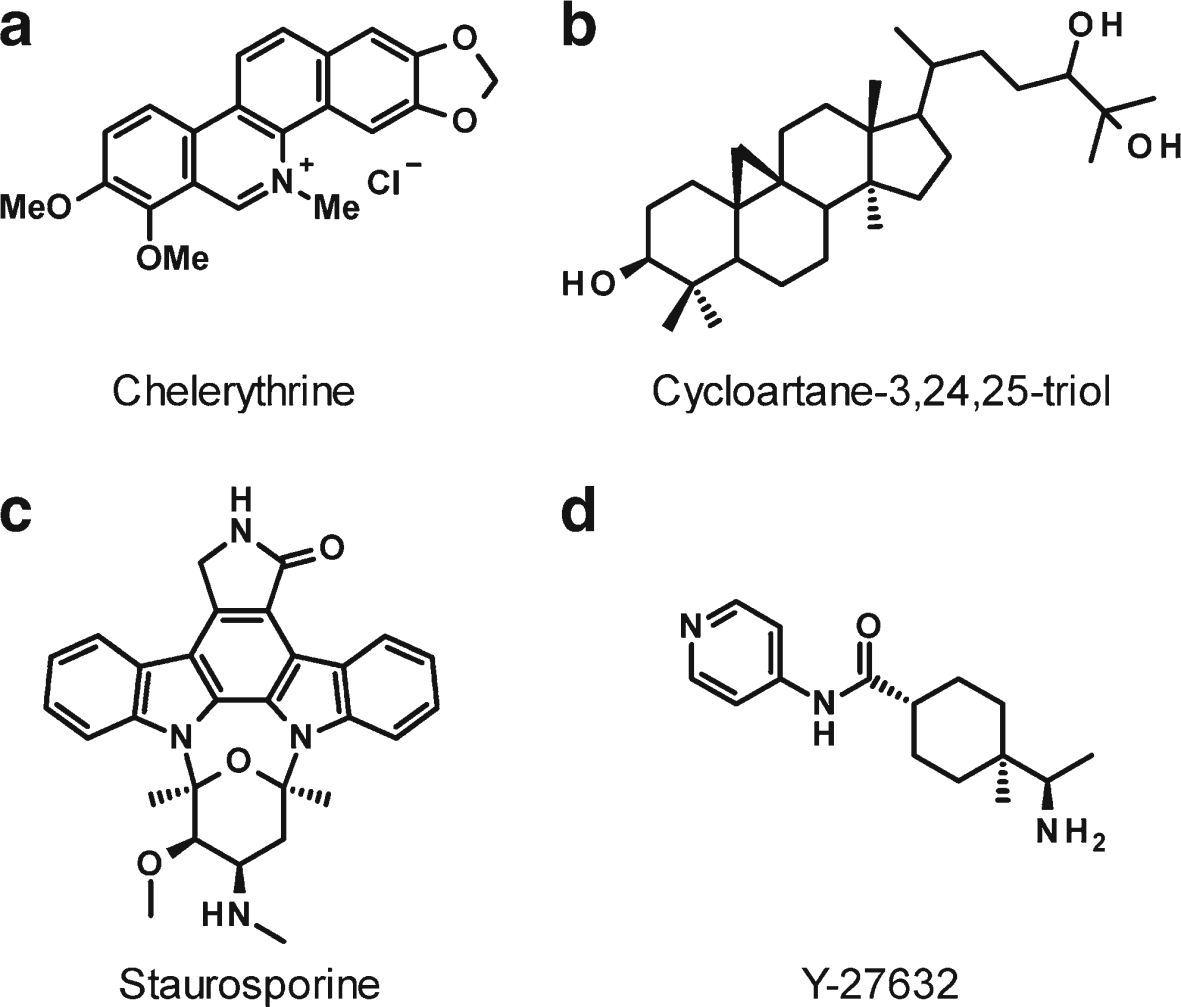
Inhibitors of MRCK described in the literature. The natural compounds **a** chelerythrine, **b** cycloartane-3,24,25-triol and **c** staurosporine have all been described as MRCK inhibitors. However, the lack of selectivity for chelerythine and staurosporine limit their value as chemical biology tools. The potency of cycloartane-3,24,25-triol in vitro and in vivo remains to be determined. **d** ROCK inhibitor Y-27632 has some effect on MRCK activity in vitro and in vivo at low micromolar concentrations, which suggests that some studies using it at concentrations in excess of 10 μM may be affecting both ROCK and MRCK function

The ROCK and MRCK kinase domains have high primary amino acid and structural homology; as a result, it is not surprising that they are able to phosphorylate many common substrates. MLC can be phosphorylated by MRCKα in vitro [7]; however, it remains to be determined whether MRCK induced elevation of MLC phosphorylation in cells is due to direct phosphorylation or the result of phosphorylation of MYPT1 [36–38] and consequent inhibition of MLC phosphatase activity (Fig. 4). Screening experiments in *Caenorhabditis elegans* revealed that MRCK and ROCK contributed to phosphorylation of MLC and MYPT1 homologues, but that a constitutively-active form of MLC could complement loss of MRCK but not ROCK [39]. These results suggested that regulation of MLC phosphorylation, possibly via MYPT1 phosphorylation, is the primary function of MRCK in *C. elegans*, while ROCK must have additional important targets. It was reported that MRCKα forms a tripartite complex with leucine repeat adaptor protein 35a (LRAP35a) and myosin 18A, which both activates kinase activity and targets MRCKα to the actin-myosin cytoskeleton to promote direct MLC phosphorylation [40]. MRCKα was also found to phosphorylate LIM kinases 1 and 2 (LIMK1 and LIMK2) [41], resulting in increased phosphorylation and inactivation of the filamentous actin severing protein cofilin [42], which would contribute to actin-myosin contractility. In addition, MRCKα induced in vitro phosphorylation of moesin [38], which links integral membrane proteins to filamentous actin, suggesting that actin-myosin contractility may be further promoted by MRCK through enhanced coupling of the cytoskeleton to the membrane. On a cell-wide basis, these phosphorylation events would collectively promote actin-myosin cytoskeleton contractility. However, the important function of MRCK in regulating actin-myosin dynamics is more likely to be confined to specific regions of a cell to contribute to distinct morphological responses.

## MRCK role in actin-myosin regulation and cell biology

There are numerous kinases that contribute to MLC phosphorylation, including MRCK and ROCK kinases. However, their contributions to MLC phosphorylation and biological functions often appear to be distinct and non-overlapping, which may be due to different sites of activation and/or recruitment. Several studies have found that blocking both MRCK and ROCK kinases is necessary for full inhibition of specific responses. The ability of ephrinB to induce endothelial cell retraction required the combined blockade of ROCK and MRCK [43]. Similarly, inhibition of ROCK and MRCK produced the largest reduction in MDA MB 231 breast cancer cell invasion into three-dimensional protein matrices [17, 37]. Genetic analysis in *C. elegans* revealed differing timing and localization of MLC phosphorylation mediated by ROCK and MRCK homologues during asymmetric division [39]. Similarly, endothelial cells were found to require MRCKβ for MLC phosphorylation that contributed to the formation of circumferential actin bundles proximal to the plasma membrane that promote the formation of linear adherens junctions and tight endothelial barriers in response to elevated cyclic AMP [44]. In contrast, MLC phosphorylation by ROCK led to the formation of radial stress fibres that promote adherens junction clustering and reduced endothelial barrier function [44]. These studies support the concept that MRCK and ROCK may share similar substrates, but differences in their activation by signalling pathways combined with dissimilarities in their subcellular localization, in basal and/or stimulated states, results in distinct responses.

The recruitment of MRCKβ to the leading edge of migrating kidney cells through association with the tight junction protein ZO-1 and active CDC42 was found to be required for polarized cell migration [45]. One way that MRCK recruited to leading edge membranes and cytoskeletal structures may promote motility is by increasing actin-myosin retrograde flow, which helps cytoskeleton-tethered transmembrane proteins, such as integrin complexes, to generate tractive forces for cell movement [40]. In addition, the actin-myosin retrograde flow induced by MRCK aids re-orientation of cell nuclei relative to microtubule-organizing centres (MTOC) to establish polarity and directionality in migrating cells [46]. The requirement for MRCK to establish polarity might also account for the requirement for MRCK to assemble matrix degrading complexes containing MT1-MMP for degradation of extracellular matrix to permit cell invasion [47]. MRCKβ was also found to contribute to the ability of invasive breast cancer cells to degrade matrix through cathepsin B expression [48]. In an organotypic model of SCC invasion, collective invasion of the SCC12 cell line through physical tracks made by carcinoma associated fibroblasts in three-dimensional protein matrices was dependent on MRCK signalling [49]. This may be due to a role for MRCK in cell polarity and motility as well as protease secretion described above, as well as contributing to cortical actin-myosin contractility that is required for maintenance of the cell collective as a unit. These results indicate that there is a significant role for MRCK in promoting cell motility and invasiveness, which acts in concert with the contributions of ROCK through independent and non-compensatory mechanisms.

## MRCK expression in cancer

**Fig. 5:**
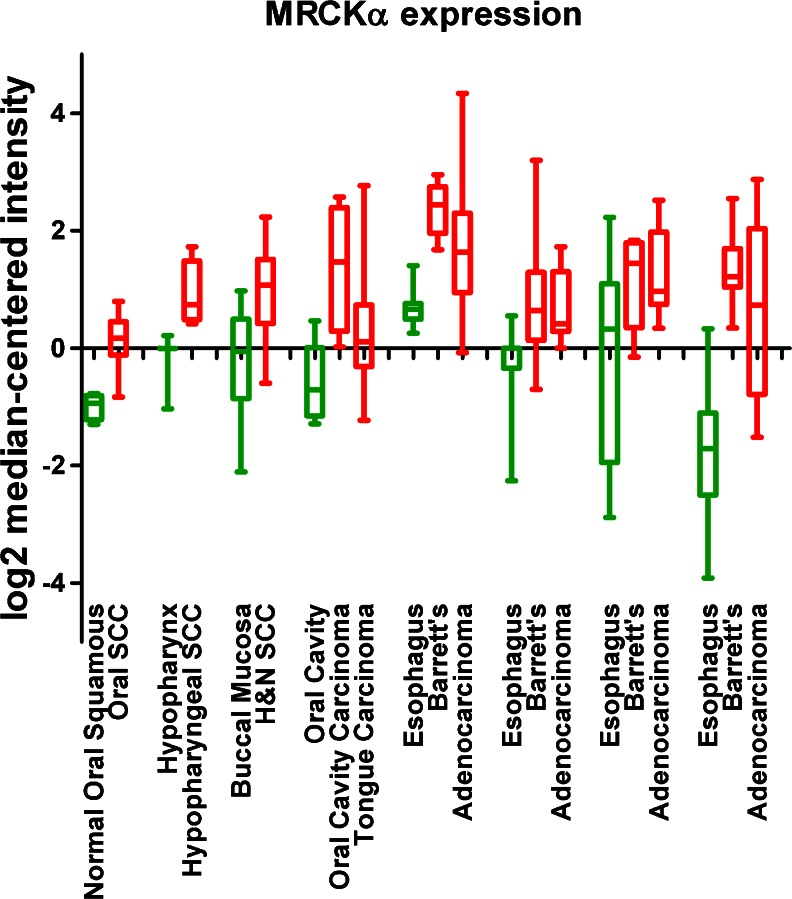
MRCKα expression in carcinomas and Barrett’s esophagus. Analysis of publicly available gene expression microarray data using Oncomine revealed significantly increased MRCKα levels (*red*) in oral, hypopharyngeal, head and neck squamous cell carcinoma, oral cavity and tongue carcinoma, Barrett’s esophagus and esophageal adenocarcinoma relative to matched normal tissue (*green*). *Box* represents upper and lower quartiles with line at median, *whiskers* indicate maximum and minimum values

Actin-myosin contractility is a key component of cell motility and is required for cancer cell invasion and for the metastatic process to occur [50]. Therefore, elevated MRCK expression could be expected to be elevated in invasive and metastatic cancer. Northern blots with a MRCKα kinase domain cDNA probe revealed relatively high levels of RNA expression in U937 histiocytic lymphoma, MDA MB 231 breast cancer, A549 lung cancer and PLB 985 myelocytic leukemia cell lines [10]. MRCKα (designated PK428) was identified in breast cancer microarrays as being part of a gene expression signature linked to poor prognosis and increased incidence of metastasis under 5 years [51]. MRCKα was also identified as being part of a seven-gene panel displaying significantly elevated expression in pancreatic adenocarcinoma compared to normal pancreas tissue [52]. Up-regulation of MRCKα expression was reported to contribute to cutaneous squamous cell carcinoma (SCC) following the repression of the Notch1 tumour suppressor [53]. Similar to this observation, analysis of publicly available gene expression microarray data using Oncomine [54] revealed that MRCKα expression is elevated in oral, hypopharyngeal, and head and neck SCC, oral cavity and tongue carcinoma, and Barrett’s esophagus compared to normal tissues (Fig. 5) [55–61]. These results indicate that there are associations between altered MRCK expression and several cancers, particularly with cancers of squamous epithelia, which may reflect roles in promoting invasion and metastasis.

## Conclusions

MRCK kinases are downstream effectors of CDC42 that play key roles in actin-myosin regulation and activities including cell adhesion, motility and invasion. Despite sharing several common substrates, ROCK and MRCK are independently regulated and undertake different functions. These differences are likely the product of large variations in subcellular localization; the array of membrane-targeting domains in MRCK, including the CDC42-binding CRIB domain, serve to concentrate the protein to actin-myosin cytoskeletal structures proximal to the plasma membrane while RhoA leads to ROCK activation throughout the cell. As a result, in several contexts, their combined inhibition is required to block specific processes, such as cancer cell invasion. Expression profiles indicate that MRCK expression may be elevated in some cancers, where they may have important roles in tumour progression. Drug targeting of MRCK, potentially in combination with ROCK, seems to be an attractive strategy to potentially inhibit cancer cell spread, although current inhibitors lack specificity that would allow this hypothesis to be rigorously tested. The development of MRCK inhibitors that were selective or also inhibit ROCK activity could be an effective strategy for reducing the spread of invasive and metastatic cancers.

## References

[1] Vicente-Manzanares M, Ma X, Adelstein RS, Horwitz AR (2009). Non-muscle myosin II takes centre stage in cell adhesion and migration. Nat Rev Mol Cell Biol.

[2] Somlyo AP, Somlyo AV (2003). Ca2+ sensitivity of smooth muscle and nonmuscle myosin II: modulated by G proteins, kinases, and myosin phosphatase. Physiol Rev.

[3] Olson MF (2008). Applications for ROCK kinase inhibition. Curr Opin Cell Biol.

[4] Madaule P, Eda M, Watanabe N, Fujisawa K, Matsuoka T, Bito H, Ishizaki T, Narumiya S (1998). Role of citron kinase as a target of the small GTPase Rho in cytokinesis. Nature.

[5] Uehata M, Ishizaki T, Satoh H, Ono T, Kawahara T, Morishita T, Tamakawa H, Yamagami K, Inui J, Maekawa M (1997). Calcium sensitization of smooth muscle mediated by a Rho-associated protein kinase in hypertension. Nature.

[6] Rath N, Olson MF (2012). Rho-associated kinases in tumorigenesis: re-considering ROCK inhibition for cancer therapy. EMBO Rep.

[7] Leung T, Chen X-QQ, Tan I, Manser E, Lim L (1998). Myotonic dystrophy kinase-related Cdc42-binding kinase acts as a Cdc42 effector in promoting cytoskeletal reorganization. Mol Cell Biol.

[8] Ng Y, Tan I, Lim L, Leung T (2004). Expression of the human myotonic dystrophy kinase-related Cdc42-binding kinase gamma is regulated by promoter DNA methylation and Sp1 binding. J Biol Chem.

[9] Pearce LR, Komander D, Alessi DR (2010). The nuts and bolts of AGC protein kinases. Nat Rev Mol Cell Biol.

[10] Zhao Y, Loyer P, Li H, Valentine V, Kidd V, Kraft AS (1997). Cloning and chromosomal location of a novel member of the myotonic dystrophy family of protein kinases. J Biol Chem.

[11] Luo L, Lee T, Tsai L, Tang G, Jan LY, Jan YN (1997). Genghis Khan (Gek) as a putative effector for Drosophila Cdc42 and regulator of actin polymerization. Proc Natl Acad Sci U S A.

[12] Tan I, Cheong A, Lim L, Leung T (2003). Genomic organization of human myotonic dystrophy kinase-related Cdc42-binding kinase α reveals multiple alternative splicing and functional diversity. Gene.

[13] Moncrieff CL, Bailey ME, Morrison N, Johnson KJ (1999). Cloning and chromosomal localization of human Cdc42-binding protein kinase beta. Genomics.

[14] Pirone DM, Carter DE, Burbelo PD (2001). Evolutionary expansion of CRIB-containing Cdc42 effector proteins. Trends Genet.

[15] Schwarz J, Proff J, Havemeier A, Ladwein M, Rottner K, Barlag B, Pich A, Tatge H, Just I, Gerhard R (2012). Serine-71 phosphorylation of Rac1 modulates downstream signaling. PLoS One.

[16] Corpet F (1988). Multiple sequence alignment with hierarchical clustering. Nucleic Acids Res.

[17] Heikkila T, Wheatley E, Crighton D, Schroder E, Boakes A, Kaye SJ, Mezna M, Pang L, Rushbrooke M, Turnbull A (2011). Co-crystal structures of inhibitors with MRCKbeta, a key regulator of tumor cell invasion. PLoS One.

[18] Tan I, Seow KT, Lim L, Leung T (2001). Intermolecular and intramolecular interactions regulate catalytic activity of myotonic dystrophy kinase-related Cdc42-binding kinase alpha. Mol Cell Biol.

[19] Elkins JM, Amos A, Niesen FH, Pike AC, Fedorov O, Knapp S (2009). Structure of dystrophia myotonica protein kinase. Protein Sci.

[20] Jacobs M, Hayakawa K, Swenson L, Bellon S, Fleming M, Taslimi P, Doran J (2006). The structure of dimeric ROCK I reveals the mechanism for ligand selectivity. J Biol Chem.

[21] Yamaguchi H, Kasa M, Amano M, Kaibuchi K, Hakoshima T (2006). Molecular mechanism for the regulation of rho-kinase by dimerization and its inhibition by fasudil. Structure.

[22] Lam LT, Pham YC, Nguyen TM, Morris GE (2000). Characterization of a monoclonal antibody panel shows that the myotonic dystrophy protein kinase, DMPK, is expressed almost exclusively in muscle and heart. Hum Mol Genet.

[23] Choi SH, Czifra G, Kedei N, Lewin NE, Lazar J, Pu Y, Marquez VE, Blumberg PM (2008). Characterization of the interaction of phorbol esters with the C1 domain of MRCK (myotonic dystrophy kinase-related Cdc42 binding kinase) alpha/beta. J Biol Chem.

[24] Loo CS, Chen CW, Wang PJ, Chen PY, Lin SY, Khoo KH, Fenton RA, Knepper MA, Yu MJ (2013). Quantitative apical membrane proteomics reveals vasopressin-induced actin dynamics in collecting duct cells. Proc Natl Acad Sci U S A.

[25] Liu X, Yu X, Zack DJ, Zhu H, Qian J (2008). TiGER: a database for tissue-specific gene expression and regulation. BMC Bioinforma.

[26] Cmejla R, Petrak J, Cmejlova J (2006). A novel iron responsive element in the 3'UTR of human MRCKalpha. Biochem Biophys Res Commun.

[27] Herbert JM, Augereau JM, Gleye J, Maffrand JP (1990). Chelerythrine is a potent and specific inhibitor of protein kinase C. Biochem Biophys Res Commun.

[28] Tan I, Lai J, Yong J, Li SF, Leung T (2011). Chelerythrine perturbs lamellar actomyosin filaments by selective inhibition of myotonic dystrophy kinase-related Cdc42-binding kinase. FEBS Lett.

[29] Yamamoto S, Seta K, Morisco C, Vatner SF, Sadoshima J (2001). Chelerythrine rapidly induces apoptosis through generation of reactive oxygen species in cardiac myocytes. J Mol Cell Cardiol.

[30] Basu P, Bhowmik D, Suresh Kumar G (2013). The benzophenanthridine alkaloid chelerythrine binds to DNA by intercalation: photophysical aspects and thermodynamic results of iminium versus alkanolamine interaction. J Photochem Photobiol B.

[31] Brunhofer G, Fallarero A, Karlsson D, Batista-Gonzalez A, Shinde P, Gopi Mohan C, Vuorela P (2012). Exploration of natural compounds as sources of new bifunctional scaffolds targeting cholinesterases and beta amyloid aggregation: the case of chelerythrine. Bioorg Med Chem.

[32] Heerding DA, Rhodes N, Leber JD, Clark TJ, Keenan RM, Lafrance LV, Li M, Safonov IG, Takata DT, Venslavsky JW (2008). Identification of 4-(2-(4-amino-1,2,5-oxadiazol-3-yl)-1-ethyl-7-{[(3*S*)-3-piperidinylmethyl]oxy}-1*H*-imidazo[4,5-*c*]pyridin-4-yl)-2-methyl-3-butyn-2-ol (GSK690693), a novel inhibitor of AKT kinase. J Med Chem.

[33] Lowe HI, Watson CT, Badal S, Toyang NJ, Bryant J (2012). Cycloartane-3,24,25-triol inhibits MRCKalpha kinase and demonstrates promising anti prostate cancer activity in vitro. Cancer Cell Int.

[34] Fabian MA, Biggs WH, Treiber DK, Atteridge CE, Azimioara MD, Benedetti MG, Carter TA, Ciceri P, Edeen PT, Floyd M (2005). A small molecule-kinase interaction map for clinical kinase inhibitors. Nat Biotechnol.

[35] Podolin PL, Callahan JF, Bolognese BJ, Li YH, Carlson K, Davis TG, Mellor GW, Evans C, Roshak AK (2005). Attenuation of murine collagen-induced arthritis by a novel, potent, selective small molecule inhibitor of IkappaB Kinase 2, TPCA-1 (2-[(aminocarbonyl)amino]-5-(4-fluorophenyl)-3-thiophenecarboxamide), occurs via reduction of proinflammatory cytokines and antigen-induced T cell proliferation. J Pharmacol Exp Ther.

[36] Tan I, Ng CH, Lim L, Leung T (2001). Phosphorylation of a novel myosin binding subunit of protein phosphatase 1 reveals a conserved mechanism in the regulation of actin cytoskeleton. J Biol Chem.

[37] Wilkinson S, Paterson HF, Marshall CJ (2005). Cdc42-MRCK and Rho-ROCK signalling cooperate in myosin phosphorylation and cell invasion. Nat Cell Biol.

[38] Nakamura N, Oshiro N, Fukata Y, Amano M, Fukata M, Kuroda S, Matsuura Y, Leung T, Lim L, Kaibuchi K (2000). Phosphorylation of ERM proteins at filopodia induced by Cdc42. Genes Cells.

[39] Gally C, Wissler F, Zahreddine H, Quintin S, Landmann F, Labouesse M (2009). Myosin II regulation during C. elegans embryonic elongation: LET-502/ROCK, MRCK-1 and PAK-1, three kinases with different roles. Development.

[40] Tan I, Yong J, Dong JM, Lim L, Leung T (2008). A tripartite complex containing MRCK modulates lamellar actomyosin retrograde flow. Cell.

[41] Scott RW, Olson MF (2007). LIM kinases: function, regulation and association with human disease. J Mol Med.

[42] Sumi T, Matsumoto K, Shibuya A, Nakamura T (2001). Activation of LIM kinases by myotonic dystrophy kinase-related Cdc42-binding kinase alpha. J Biol Chem.

[43] Groeger G, Nobes CD (2007). Co-operative Cdc42 and Rho signalling mediates ephrinB-triggered endothelial cell retraction. Biochem J.

[44] Ando K, Fukuhara S, Moriya T, Obara Y, Nakahata N, Mochizuki N (2013). Rap1 potentiates endothelial cell junctions by spatially controlling myosin II activity and actin organization. J Cell Biol.

[45] Huo L, Wen W, Wang R, Kam C, Xia J, Feng W, Zhang M (2011). Cdc42-dependent formation of the ZO-1/MRCKbeta complex at the leading edge controls cell migration. EMBO J.

[46] Gomes ER, Jani S, Gundersen GG (2005). Nuclear movement regulated by Cdc42, MRCK, myosin, and actin flow establishes MTOC polarization in migrating cells. Cell.

[47] Fisher KE, Sacharidou A, Stratman AN, Mayo AM, Fisher SB, Mahan RD, Davis MJ, Davis GE (2009). MT1-MMP- and Cdc42-dependent signaling co-regulate cell invasion and tunnel formation in 3D collagen matrices. J Cell Sci.

[48] Rafn B, Nielsen CF, Andersen SH, Szyniarowski P, Corcelle-Termeau E, Valo E, Fehrenbacher N, Olsen CJ, Daugaard M, Egebjerg C (2012). ErbB2-driven breast cancer cell invasion depends on a complex signaling network activating myeloid zinc finger-1-dependent cathepsin B expression. Mol Cell.

[49] Gaggioli C, Hooper S, Hidalgo-Carcedo C, Grosse R, Marshall JF, Harrington K, Sahai E (2007). Fibroblast-led collective invasion of carcinoma cells with differing roles for RhoGTPases in leading and following cells. Nat Cell Biol.

[50] Olson MF, Sahai E (2009). The actin cytoskeleton in cancer cell motility. Clin Exp Metastasis.

[51] van’t Veer LJ, Dai H, van de Vijver MJ, He YD, Hart AAM, Mao M, Peterse HL, van der Kooy K, Marton MJ, Witteveen AT (2002). Gene expression profiling predicts clinical outcome of breast cancer. Nature.

[52] Balasenthil S, Chen N, Lott ST, Chen J, Carter J, Grizzle WE, Frazier ML, Sen S, Killary AM (2011). A migration signature and plasma biomarker panel for pancreatic adenocarcinoma. Cancer Prev Res.

[53] Lefort K, Mandinova A, Ostano P, Kolev V, Calpini V, Kolfschoten I, Devgan V, Lieb J, Raffoul W, Hohl D (2007). Notch1 is a p53 target gene involved in human keratinocyte tumor suppression through negative regulation of ROCK1/2 and MRCK-alpha kinases. Genes Dev.

[54] Rhodes DR, Yu J, Shanker K, Deshpande N, Varambally R, Ghosh D, Barrette T, Pandey A, Chinnaiyan AM (2004). ONCOMINE: a cancer microarray database and integrated data-mining platform. Neoplasia.

[55] Pyeon D, Newton MA, Lambert PF, den Boon JA, Sengupta S, Marsit CJ, Woodworth CD, Connor JP, Haugen TH, Smith EM (2007). Fundamental differences in cell cycle deregulation in human papillomavirus-positive and human papillomavirus-negative head/neck and cervical cancers. Cancer Res.

[56] Schlingemann J, Habtemichael N, Ittrich C, Toedt G, Kramer H, Hambek M, Knecht R, Lichter P, Stauber R, Hahn M (2005). Patient-based cross-platform comparison of oligonucleotide microarray expression profiles. Lab Invest.

[57] Ginos MA, Page GP, Michalowicz BS, Patel KJ, Volker SE, Pambuccian SE, Ondrey FG, Adams GL, Gaffney PM (2004). Identification of a gene expression signature associated with recurrent disease in squamous cell carcinoma of the head and neck. Cancer Res.

[58] Kim SM, Park YY, Park ES, Cho JY, Izzo JG, Zhang D, Kim SB, Lee JH, Bhutani MS, Swisher SG (2010). Prognostic biomarkers for esophageal adenocarcinoma identified by analysis of tumor transcriptome. PLoS One.

[59] Wang S, Zhan M, Yin J, Abraham JM, Mori Y, Sato F, Xu Y, Olaru A, Berki AT, Li H (2006). Transcriptional profiling suggests that Barrett’s metaplasia is an early intermediate stage in esophageal adenocarcinogenesis. Oncogene.

[60] Hao Y, Triadafilopoulos G, Sahbaie P, Young HS, Omary MB, Lowe AW (2006). Gene expression profiling reveals stromal genes expressed in common between Barrett’s esophagus and adenocarcinoma. Gastroenterology.

[61] Kimchi ET, Posner MC, Park JO, Darga TE, Kocherginsky M, Karrison T, Hart J, Smith KD, Mezhir JJ, Weichselbaum RR (2005). Progression of Barrett’s metaplasia to adenocarcinoma is associated with the suppression of the transcriptional programs of epidermal differentiation. Cancer Res.

